# Evaluation and forecasting methods to estimate number of patients with non-Hodgkin lymphoma: a systematic literature review

**DOI:** 10.1186/s12963-025-00415-8

**Published:** 2025-10-21

**Authors:** Dan Lin, Changxia Shao, James R. Rogers, Mehmet Burcu, Helmneh M. Sineshaw

**Affiliations:** 1 Merck & Co., Inc, Rahway, NJ 07065 USA; 2 Merck & Co., Inc, West Point, PA 19486 USA

**Keywords:** Projection method, Evaluation, Validation, Incidence, Prevalence, Number of treatment eligible patients, Cancer, Non-Hodgkin lymphoma, Diffuse large B-cell lymphoma

## Abstract

**Background:**

Non-Hodgkin lymphoma (NHL) is the most common hematologic cancer in the US. Validated projections of NHL cases are important for various stakeholders. The study aimed to identify and characterize methods forecasting NHL incidence, prevalence, and number of treatment eligible patients with NHL by line of therapy (LoT). In addition, methods evaluating the performance of cancer forecasting methods were also identified and utilized in selecting the most robust projection method applicable to NHL disease setting.

**Methods:**

A comprehensive search was conducted in MEDLINE and EMBASE databases, covering January 2002 to April 2024 for English-language studies reporting methods evaluating cancer count estimation and NHL projection methods. Study characteristics were extracted and described. Criteria was developed to identify the most appropriate methods for evaluating projection methods. The identified methods of evaluation were then adopted to measure the accuracy of NHL projection methods.

**Results:**

Twenty-nine articles met the inclusion criteria for methods of evaluation, with 58.6% evaluating projection methods through calculating relative difference between observed and predicted case numbers. The most appropriate methods found for evaluating cancer incidence and prevalence projection were the average absolute relative deviation (AARD) and percent variation (VAR%), respectively. These methods were applied to projection methods identified through literature review to determine the robust method to project incidence and prevalence. Among twenty-six articles met the inclusion criteria for NHL projection methods, the joinpoint regression model was determined as the most robust method for projecting NHL incidence in the US, with the lowest AARD (1.6%). The projection method with assumptions of a 52.8% cure rate, a cure beginning ten years post-diagnosis, and all surviving patients cured after 20 years was identified as the most robust method for projecting NHL prevalence, with the lowest VAR% (8.3%). Unfortunately, due to the limited number and quality of studies, no robust method was identified for projecting the number of treatment-eligible NHL patients by LoT.

**Conclusion:**

This review identified the most appropriate method of evaluating projection methods, and identified methods for projecting NHL incidence and prevalence in the US. Nevertheless, further research is needed to validate and project the number of treatment-eligible NHL patients by LoT.

**Supplementary Information:**

The online version contains supplementary material available at 10.1186/s12963-025-00415-8.

## Introduction

Non-Hodgkin lymphoma (NHL) is a type of cancer that arises from the cells in the lymphatic system [1] and remains the most common hematologic cancer in the United States (US) [2]. The number of incident cases of NHL increased during 1999–2019 because of the growing and aging US population, but declined during 2020–2021 due to the delayed cancer screenings and diagnoses caused by COVID-19 pandemic [3]. In 2024, it is estimated that there will be a total of 80,620 new cases of NHL, with 44,590 in men and 36,030 in women [4]. Treatment options for NHL are changing rapidly with major variations by subtype and line of therapy (LoT).

Diffuse large B cell lymphoma (DLBCL) is the most common subtype of NHL in the US, accounting for about 30% of all cases of NHL [5]. While more than 60% of DLBCL patients are cured with first line therapy, about 10–15% have primary refractory disease and an additional 20–25% will relapse within the first 2 years of diagnosis [6]. Notably, the introduction of promising therapies, such as monoclonal antibodies (mAbs), chimeric antigen receptor T-cell therapy (CAR-T), and antibody-drug conjugates (ADCs) has demonstrated efficacy in treating DLBCL in recent years [7–9]. Mantle cell lymphoma (MCL), another subtype of NHL, consists of 3–10% of all NHL cases in the US [10]. Historically, MCL had a poor prognosis with a median overall survival (OS) of 4–5 years [11]. Recent advancements in treatment, such as immunochemotherapy induction and the use of 2nd generation BTK inhibitors (acalabrutinib and zanubrutinib), have substantially improved outcomes for MCL patients [12, 13].

Projecting the incidence, prevalence, and number of treatment eligible patients with NHL is important for various stakeholders, such as healthcare providers, policy makers, and drug developers. By forecasting these numbers, stakeholders can better understand the burden of NHL, identify unmet need, and anticipate the resources needed for drug discovery, drug development, and access to treatment. Several projection methods are used to forecast the incidence [14–16], prevalence [6, 17], and number of treatment eligible patients with NHL by LoT [6, 18]. However, there is significant variability in the estimated number of NHL cases across different projection methods, and there is also a lack of evaluation and summary of methods that could validate the performance of these projection methods. The reliability and validity of these projection methods remains unclear. Therefore, it is necessary to conduct a comprehensive review and assessment for methods of evaluation and forecasting methods.

The aim of this study was to (1) identify and summarize methods that have been used in assessing the performance of cancer count estimation methods, (2) identify and characterize methods for estimating/forecasting the incidence, prevalence, and number of treatment eligible patients with NHL, focusing on DLBCL and MCL by LoT, and (3) apply the selected method in aim 1 to assess estimation/forecasting methods identified in aim 2, and to identify the most robust method for NHL projection.

## Methods

The process used to achieve study aims consists of three steps. In Step 1, two systematic literature reviews (SLRs) were conducted. The first SLR was to identify evaluation methods to assess any cancer forecasting methods. The second SLR was to identify forecasting methods for numbers of NHL patients. In Step 2, the quality of evaluation methods was evaluated to determine the most appropriate one for assessing forecasting methods. In Step 3, the selected evaluation method from Step 2 was applied to measure the accuracy of methods for forecasting numbers of NHL patients.

### SLRs of evaluation methods and forecasting methods

#### Criteria for study inclusion/exclusion

According to a typology and guidance for systematic reviewers proposed by Munn et al. [19], which is based on the *guide to the contents of a Cochrane Methodology protocol and review* [20], the SDMO (*S*tudy Design, *D*ata, *M*ethods, and *O*utcomes) criteria are best suited for methodological reviews. Therefore, we employed SDMO to align with the research objective and facilitate the implementation of appropriate search strategy.

Studies eligible for aim 1 were those that reported any methods used to validate/evaluate the estimation or projection of incidence, prevalence, or number of treatment eligible patients for any cancer (excluding non-melanoma skin cancer) at the national or regional level. Studies eligible for aim 2 were those that reported at least one method for estimating or forecasting the incidence, prevalence, or number of treatment eligible patients with NHL, especially DLBCL and MCL by LoT in any population-based data. The identification and selection of eligible studies were based on the SDMO criteria (**Additional File 1**). Only studies published in English from January 2002 to May 2024 were included. Year 2002 was chosen to align with the release of International Classification of Disease for Oncology, 3rd Edition (ICD-O-3) coding of cancer cases.

#### Search strategy

Relevant literature was retrieved in June 2024 using a combination of controlled vocabularies and keyword search terms in two databases: (1) EMBASE, and (2) PubMed. In evaluation methods identification (aim 1), search terms comprised synonyms of “forecasting”, “validity”, “cancer”, “epidemiology” or “incidence” or “prevalence” or “treatment eligible patients”. In projection methods identification (aim 2), search terms comprised synonyms of “forecasting”, “non-Hodgkin lymphoma”, “epidemiology” or “incidence” or “prevalence” or “treatment eligible patients”. The complete search strategy is provided in **Additional File 2**. References of included studies were additionally reviewed as supplemental sources that could potentially fulfill our eligibility criteria.

#### Study selection

Following the pre-defined SDMO criteria, all publications were screened by one reviewer based on abstract and title in the web app “Covidence”, a software designed to support title and abstract screening for healthcare research and has demonstrated good alignment with user requirement [21]. Uncertainties were resolved with input from the co-authors, as necessary. Publications that did not match the SDMO criteria were excluded at this stage. Duplicates of publications due to overlap in the coverage of databases were also excluded in the abstract/title screening.

**Fig. 1:**
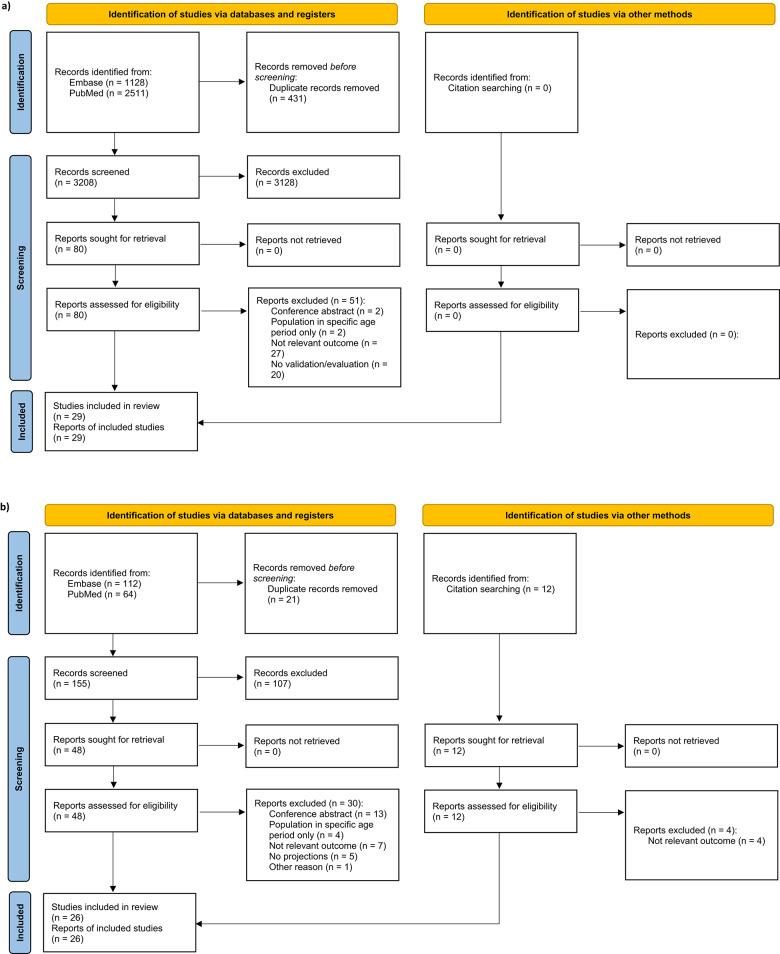
PRISMA flowchart for **a**) studies reporting evaluation methods, **b**) studies reporting projection methods

Publications included after screening of the title and abstract were then proceeded to full text screening. The single reviewer conducted the full-text review and addressed uncertainties with input from co-authors. Any publications excluded during the full text screen stage were accompanied by exclusion reasons. All studies included after the full text screen were retained for data extraction. For supplemental search, eligible studies were identified through hand-searches of the reference lists of publications that meet the eligibility criteria in the full text review. The flow diagrams from Preferred Reporting Items for Systematic Reviews and Meta-Analyses (PRISMA) were completed during the literature search (Fig. 1).

#### Quality assessment of included studies

The quality assessment of all included studies was performed using a prespecified checklist, which was previously adopted in a systematic review of methods estimating colorectal cancer incidence [22]. This checklist was developed based on the Joanna Briggs Institute Critical Appraisal tool for prevalence studies [23] and the Appraisal tool for Cross-Sectional Studies [24]. Specifically, relevant criteria from each tool were selected to create a 10-item checklist for quality assessment. Each item was scored as 1 if it was demonstrated in the study, or 0 if it was not demonstrated or unclear. An overall quality score was then calculated for each study. The quality appraisal checklist is presented (**Additional File 3a**).

#### Data collection and data synthesis

The information for studies reporting evaluation methods (aim 1) and projection methods (aim 2) was systematically extracted using two tables respectively to summarize key study characteristics and methodologies. The following data were captured in both tables: published sources (first author, publication year, title, journal/proceeding), study design (study objective, data source, data coverage, country, observation period, lengths of years projected, cancer site, projection outcome), the name and detailed description of projection methods, main conclusion, software for projection, and study limitation. The table for evaluation methods (aim 1) additionally collected information including lengths of years validated/evaluated, validation/evaluation outcome, name and detailed description of evaluation methods, and software used for validation/evaluation. Publications missing any of the above information were classified as “not reported” in the table. Studies that utilized the same evaluation method were grouped together when summarizing the statistical metrics and limitations. Similarly, studies using different projection methods were classified.

### Identifying the most appropriate method to evaluate estimation/projection methods

To determine the most appropriate method for evaluating the performance of cancer projections, the quality of each method was evaluated. A set of criteria was established based on the Guidelines for Accurate and Transparent Health Estimates Reporting (GATHER) [25] and general principles of quality for articles (Table 1). Each criterion was assigned a weighted score according to how well each study reporting the method aligned with the descriptions described in Table 1. The total score for each study was calculated by summing the score of each criterion. The method from the study with the highest score would be used to identify the robust methods for NHL projection.

**Table 1:** Criteria for assessing the quality of validation/evaluation methods

Criteria	Description	Weighted score
**Criteria for assessing the quality of any evaluation methods**		
**Evidence for data quality**	Report any details about the quality of database that were used to generate projections.	0 - No evidence; 1 - Study indicated that the database is high quality without reference citation or data quality assessment; 2 - Study indicated that the database is high quality by citing a reference for previously conducted research (e.g., data validation or completeness assessment) as evidence of database data quality, or by presenting the data quality assessment in the context.
**Coverage of data source**	Report any coverage of database that was used to generate projections.	0 - No data coverage mentioned; 1 - Study indicated that the database is subnational or national without coverage reporting; 2 - Study indicated that the database is subnational or national by reporting the % of the national population covered by the data source.
**Long-term validation**	The validation period for cancer projection is ≥ 10 yrs.	1 - Years of validation < 10 yrs; 2 - Years of validation ≥ 10 yrs.
**Observed/reference data for validation**	Report the observed/reference data which is used to compare with projected values.	0 - No source of observed/reference data reported; 1 - Using previous published lit or RW sources other than certified cancer registries as sources for observed/reference data; 2 - Using certified cancer registries as observed/reference data; 3 - Using certified cancer registries as observed/reference data and adjusting reporting delays.
**Statistical metric/indicator for validation**	Report the statistical equation and indicator that is used to measure the accuracy of projected values.	0 - No equation or indicator reported; 1 - Report the indicator for cancer validation but did not present the statistical equation for indicator calculation; 2 - Report the statistical equation and indicator for cancer validation.
**Methods sufficiently described to enable them to be replicated**	Report the statistical methods to validate the accuracy of projected values sufficiently.	0 - Unable to replicate the study based on methods description; 2 - Methods sufficiently described to enable them to be replicated.
**Missing data assessment**	Report missing data assessment and analysis.	0 - No missing data assessment; 1 - Report handling of missing data.
**Statistical software information**	Report software information for validation.	0 - No software reported for validation; 1 - Study reported software information for validation.
**Criteria for assessing the quality of methods evaluating cancer incidence projection specifically**		
**Definition of cancer incidence**	Report the cancer sites/types and the classification system for cancer types.	0 - No cancer classification system reported; 1 - Report the use of ICD-O/ICD or other cancer classification system but without describing codes for each cancer; 2 - Report the use of ICD-O/ICD or other cancer classification system and provide codes for each cancer.
**Criteria for assessing the quality of methods evaluating cancer prevalence projection specifically**		
**Definition of cancer prevalence**	Report the definition of cancer prevalence.	0 - No clear definition reported; 1 - Report the definition of cancer prevalence (e.g., complete prevalence, limited time interval prevalence).
**Criteria for assessing the quality of methods evaluating Tx-eligible patients count projection specifically**		
**Definition of Tx eligible patients**	Report the definition of treatment eligible cancer patients.	0 - No clear definition reported; 1 - Report the definition of treatment eligible patients by LoT; 2 - Report the treatment regimen for treatment eligible patients by LoT.

ICD-O = International Classification of Diseases for Oncology, 3rd Edition; Tx = treatment, LoT = line of therapy; RW = real-world

### Identifying robust methods for NHL cases estimation/projection

#### Evaluation of methods projecting NHL incidence

The method used in Liu et al. [26] was identified as the most appropriate method for validating cancer incidence estimation/projection methods. Subsequently, this method was applied to evaluate the performance of methods projecting NHL incidence. In this evaluation method, the prediction error was defined as the difference between observed and projected NHL incidence. The absolute relative deviation (ARD) was defined as the ratio of the absolute prediction error to the observed NHL incidence plus a small positive constant c for each year of diagnosis. Adding an arbitrary small positive constant c (c = 0.01) to the observed incidence was done to avoid a division by 0. Then, the average ARD (AARD) was calculated as:

$$\:AARD=\frac{\sum\:\left(\frac{\left|Et\:-Ot\right|}{c+Ot}\right)}{t},\,t=1,2,...,n$$

where E_t_ = projected incidence value at year t, O_t_ = observed incidence value at year t, t = year of diagnosis, AARD is interpreted as the average percentage deviation of the predicted value from the observed value over the projection window [26]. In addition, the median ARD (MARD) was measured to account for potential outliers in the ARD in a certain year of diagnosis. Within the same country, the lowest AARD and MARD values indicated the best performance of the method projecting the incidence of NHL in that country.

Projected NHL incidence values from the beginning of projection period to the latest year where observed NHL incidence values available were extracted from eligible studies in aim 2. The observed NHL incidence values were the most recently released observed values which could be compared with the projected NHL values in corresponding countries or regions. These observed NHL incidence values were collected from sources including NORDCAN [27] for the Nordic countries, National Program of Cancer Registries (NPCR) [28] for the US, Cancer Research UK [29] and Northern Ireland Cancer Registry [30] for the UK, Manitoba Cancer Registry [31] for Canada, French Network of Cancer Registries (FRANCIM) [32], National Agency for Cancer Registration for Switzerland [33], National Cancer Registry for Lebanon [34], International Agency for Research on Cancer (IRAC) [35] for Czech Republic, Australian Institute of Health and Welfare (AIHW) [36], and Korea National Cancer Incidence Database (KNCI DB) [37].

#### Evaluation of methods projecting DLBCL prevalence

The method used in Francisci et al. [38] was identified as the most appropriate method for validating cancer prevalence projection methods. The literature review in part 1 identified one study projecting the prevalence of NHL in Czech Republic [18] and two studies projecting the prevalence of DLBCL in the US [6, 17]. Unfortunately, we were unable to locate verifiable sources reporting the observed NHL prevalence in Czech Republic. Therefore, this paper was excluded from consideration. Instead, the method used in Francisci et al. was applied to evaluate the performance of methods projecting DLBCL prevalence in the US. In this method, percent variation between the observed NHL prevalence and projected NHL prevalence (VAR%) was calculated to measure the accuracy of the projected DLBCL prevalence with different projection methods [38]. The VAR% was defined as:

$$\:VAR\%=(Observed-Proj)/Proj$$

where Observed = limited duration prevalence extracted from SEER*Stat software, Proj = projected prevalence extracted from two eligible studies forecasting the prevalence of DLBCL The VAR% value that was closest to zero indicated the best performance of the method projecting the prevalence of DLBCL.

## Results

### Characteristics of studies reporting methods of validation/evaluation

As shown in PRISMA diagram (Fig. 1a), the systematic search yielded 3,639 records. After removal of duplicates, 3208 were screened at the title and abstract stage and 3128 were excluded. The remaining 80 full-text articles were retrieved and reviewed for eligibility. Of these, 29 were included for methods of validation/evaluation: 24 (82.8%) articles validated/evaluated methods projecting incidence [26, 39–61], 3 (10.3%) validated/evaluated methods projecting prevalence [38, 62, 63], and 2 (6.9%) articles validated/evaluated methods estimating or projecting number of treatment-eligible cancer patients [64, 65] (Table 2a).

**Table 2:** Summary of study characteristics

a) Studies reporting validation/evaluation methods	
**Country/Region, n (%)**	**Total studies (N=29)**
Multi-country	6 (20.7)
Europe	9 (31.0)
US	6 (20.7)
Asia	3 (10.3)
Canada	2 (6.9)
Australia	2 (6.9)
Brazil	1 (3.4)
**Outcomes reported**,** n (%)**	**Total studies (N = 29**
Validation of projected Incidence	24 (82.8)
Validation of projected prevalence	3 (10.3)
Validation of projected number of treatment eligible patients	2 (6.9)
**Cancer sites**,** n (%)**	**Total studies (N = 29)**
Multi-cancer	23 (79.3)
1) include NHL	13 (44.8)
2) Not include NHL	10 (34.5)
Lung cancer	2 (6.9)
Prostate cancer	1 (3.4)
Thyroid cancer	1 (3.4)
Colorectal cancer	1 (3.4)
Multiple myeloma	1 (3.4)
**No. of years validated**,** n (%)**	**Total studies (N = 29)**
1 yrs	8 (27.6)
>1 - <10 yrs	11 (37.9)
≥ 10 yrs	10 (34.5)
**Methods for incidence validation**,** n (%)**	**Total studies (N = 24)**
Comparison between observed data and projected values	18 (75.0)
Comparison between delay-adjusted observed data and projected values	3 (12.5)
Comparison between projected values with different projection models	2 (8.3)
Decision tree in Canproj	1 (4.2)
**Methods for prevalence validation**,** n (%)**	**Total studies (N = 3)**
Comparison between observed data and projected values	2 (66.7)
Comparison between projected values with different projection models	1 (33.3)
**Methods for the number of patients validation**,** n (%)**	**Total studies ** **(N = 2)**
Compared to published literature and real-world sources	2 (100.0)

**b) Studies reporting projection methods**	
**Country/Region**,** n (%)**	**Total studies ** **(N = 26)**
Asia	8 (30.8)
US	7 (26.9)
Europe	5 (19.2)
Africa	2 (7.7)
Canada	1 (3.8)
Australia	1 (3.8)
Multi-country	2 (7.7)
Outcomes reported*,** n (%)**	**Total studies ** **(N = 26)**
Incidence	25 (96.2)
prevalence	3 (11.5)
Number of treatment eligible patients	2 (7.7)
**NHL subtypes**,** n (%)**	**Total studies ** **(N = 26)**
NHL, general	24 (92.3)
DLBCL	2 (7.7)
**Observation period used for projection**,** n (%)**	**Total studies ** **(N = 26)**
Less than 10 yrs	4 (15.4)
10–19 yrs	10 (38.5)
20 yrs or more	12 (46.2)
**No. of years projected**,** n (%)**	**Total studies ** **(N = 26)**
1 yr	10 (38.5)
>1 - ≤10 yrs	8 (30.8)
>10 yrs	8 (30.8)
Projection methods for incidence*,** n (%)**	**Total studies ** **(N = 25)**
Age-period-cohort model	12 (48.0)
Joinpoint regression model	8 (32.0)
Constant incidence rate assumption method	5 (20.0)
Other generalized linear model	4 (16.0)
Other methods	2 (8.0)
**Validation of projections methods**,** n (%)**	**Total studies ** **(N = 26)**
Study compared the projected values with the observed data beyond the period included in model fitting	5 (19.2)
Study indicated that the model was tested and validated in other papers	2 (7.7)
No validation	19 (73.1)
**Statistical software**,** n (%)**	**Total studies ** **(N = 26)**
Study reported software information for NHL projection*	18 (69.2)
1) SEER	6 (23.1)
2) SAS	2 (7.7)
3) R	8 (30.8)
4) Joinpoint	7 (26.9)
5) Others	3 (11.5)
Study not reported software information	8 (30.8)

*Number of studies are not mutually exclusive

NHL = non-Hodgkin lymphoma; DLBCL = diffuse large B cell lymphoma; SEER = Surveillance, Epidemiology, and End Results Program

Of the 29 articles, 10 (34.5%) articles validated/evaluated the projection period of 10 years or more, while 8 (27.6%) articles only validated/evaluated the projection of a single year. The most common method for incidence and prevalence validation/evaluation was the comparison between observed data and projected values (75.0% of incidence articles, and 66.7% of prevalence articles). For articles that focused on validating/evaluating of methods for projecting the number of treatment-eligible patients (*n* = 2), comparisons with earlier studies and real-world sources (e.g., Kantar database) were used as the validation/evaluation method. Overall, the quality of these 29 studies was modest, with three studies meeting all 10 criteria of high quality (**Additional File 3b**). A detailed information of study characteristics is presented (**Additional File 4a**).

The detailed description of statistical metrics or indicators used for evaluation of estimation/projection methods were summarized (Table 3). Most studies (17 of 29, 58.6%) evaluated the projection methods by calculating the relative difference between observed and predicted case numbers. In some studies, the relative difference was also named as relative error, percentage error, relative deviation, or relative bias, all of which convey the same concept as relative difference. Eight studies (27.6%) used other statistical metrics or indicators for validation/evaluation such as the sum of squared deviations, the average absolute relative deviation between observed and predicated number of cases, normalized mean square error, and absolute difference between observed and predicted number of cases. Four studies (13.8%) did not report any statistical metric or indicator used for validation/evaluation.

**Table 3:** Summary of statistical metrics or indicators used for validation/evaluation

Method for validation/evaluation	Statistical metrics	Note	Limitations	Reference
**a) incidence validation**				
**Relative difference** between observed and predicted number of cases	$RD=100\,{\times}\frac{\left|Et\:-Ot\right|}{Ot},\,t=1,2,...,n$	RD = relative difference, E = predicted value, O = observed value, t = year of dx, usually average the RD over each single year of the prediction window * In some articles, RD may call different names, e.g., relative error, percentage error, relative deviation, relative bias	1. RD may not be well-defined or meaningful when observed or predicted values are close to zero.	Moller 2003 Mitton 2011 Rutherford 2012 Uhry 2013 [1] Uhry 2013 [2] Katanoda 2014 Antoni 2016 Earnest 2019 Demers 2020 Uhry 2020 Cheng 2021 Redondo-Sanchez 2021 Luo 2021 Li 2022 Nguyen 2022
The **average absolute relative deviation** between delay-adjusted observed and predicted number of cases The **median absolute relative deviation**	$\:AARD=\frac{\sum\:\left(\frac{\left|Et\:-\overline{O}t\right|}{c+\overline{O}t}\right)}{t},\,t=1,2,...,n$ $MARD=Median\left(\frac{\left|Et\:-\overline{O}t\right|}{c+\overline{O}t},\,t=1,2,...,n\right)$	AARD = average absolute relative difference, MARD = median absolute relative deviation, c = a small positive constant c (e.g., c=0.01), E = predicted value, Ō = delay-adjusted observed value, t = year of dx, AARD is the average percentage deviation of the predicted value from the observed value over the prediction window * Adding an arbitrary small positive constant c to the observed counts was done to avoid a division by 0.	1. As a mean-based measure, AARD is sensitive to outliers and may be influenced by extreme values; 2. Both MARD and AARD only consider the absolute value of the RD and do not consider the direction of the deviations.	Zhu 2012 Liu 2021 Miller 2021
Sum of **squared deviations** (square of the estimated minus observed counts) for each state	$SSD =\sum\:\left(\left(ES-OS\right)^2\right),\,t=1,2,...,n$	SSD = sum of squared deviations, E = predicted value, O=observed value, s = number of state, usually average the SSD over the year of prediction	1. SSD is sensitive to extreme values or outliers, as the squared differences magnify their impact.	Pickle 2007
**Normalized mean square error** over the projection period	$NMSR=\frac{\sum\:\left(\frac{\left(Et\:-Ot\right)^2}{var(O)}\right)}{t},\, t=1,2,...,n$ $Var(O) =\frac{\left(\sum(Ot-\mu)^2\right)}{t},\,t=1,2,...,n$	NMSR = normalized mean square error, E = predicted value, O = observed value, var = the variance of observed value, t = year of dx, = mean of observed value, * NMSE quantified the overall magnitude of the projection error in the given period	1. NMSR is sensitive to outliers; 2. NMSR has interpretation challenges.	Li 2022
**Absolute difference** between observed and predicted number of cases	$AD=|Et-Ot|,\,t=1,2, ....,n$	AD = absolute difference, E = predicted value, O = observed value, t = year of dx	1. Absolute difference is not normalized with respect to the scale or range of the values being compared and may be influenced by the inherent magnitude of the values themselves; 2. The absolute value is lack of directionality (i.e., whether one value is higher or lower than the other).	Luo 2022
**Root mean square error** (square root of the average of the squared differences between the predicted and observed values)	$RMSE=\sqrt\frac{\sum((Et-Ot)^2)}{t},\,t=1,2,...,n$	RMSE = root mean square error, E = predicted value, O = observed value, t = year of dx * A low RMSE in sample value indicates a good average fit of the model	1. RMSE is sensitive to outliers; 2. RMSE has interpretation challenges.	Bouzon Nagem Assad 2024
Scoring rules - the logarithmic score (LogS)	$Log\,s=-log\,(px)$	is the predicted probability mass at the observed mass at the observed count x	1. Scoring rules are hard to interpret.	Clèries 2012
Scoring rules - the Dawid–Sebastiani score (DSS)	$DDS=\left(x-\frac{{\mu}p}{{\sigma}p}\right)^2+2log\,({\sigma}p)$	and p are the mean and standard deviation of the predicted distribution		
Scoring rules - the ranked probability score (RPS)	$RPS=\sum\nolimits_{k=o}^\infty\{{Pk-1(x\leq}\,k)\}^2$	Pk is the predicted cumulative distribution function		
Operating characteristics for evaluation (bias, coverage and precision)	**Coverage**: the fraction of projections lying with the 95% (equal tailed) credibility band; **Bias**: If the observed value = predicted value, Bias = 0, otherwise Bias = |O - E|/O; **Precision**: Posterior standard deviations	The operation was designed to evaluate an R package (incAnalysis)	1. Not straightforward and not user friendly. Lack description.	Knoll 2020
**b) Prevalence validation**				
Percent variation between ESTIMATE and PROJ	$VAR\%=(ESTIMATE -PROJ)/PROJ$	VAR% = percent variation, ESTIMATE = number of complete prevalence cases estimated from COMPREV software (based on limited-duration prevalence extracted from SEER*Stat software), PROJ = projected number of complete prevalent cases	1. VAR% may not be well-defined or meaningful when ESTIMATE or PROJ values close to zero.	Francisci 2023
The **weighted average percent relative difference** in absolute value between estimated and observed 20-yr number of prevalence cases	$APRD=100\,\times{\sum}_{r} \frac{|E20,r-020,r|}{020,r}Wr$	APRD = average percent relative difference, Registry-specific proportions of cancer cases (wr) were used as weights	1. Not clear for weights; 2. Hard to interpret.	Demuru 2023

### Appropriate methods to evaluate methods estimating/projecting cancer counts

Pre-defined criteria in Table 1 were employed to determine the most appropriate method for validating/evaluating the performance of cancer projection methods. Specifically, the method with statistical metric that calculated the average absolute relative deviation between observed and projected incidence, as used in Liu et al. [26], received the highest score among methods used for validation of methods forecasting incidence (Table 4a); thus, this method was determined as the most appropriate one for validating the projection methods of cancer incidence. Similarly, the method with statistical metric that calculated the percent variation between observed and projected prevalence, as used in Francisci et al. [38], was identified as the most appropriate one for validating the projection methods of cancer prevalence (Table 4b).

The methods used in two articles validating the methods projecting number of treatment-eligible cancer patients did not receive a good score in quality assessment of methods, with both being considered as suboptimal quality (Table 4c). Therefore, no appropriate method was identified to validate the methods projecting number of treatment eligible cancer patients.

**Table 4:** The quality score of validation/evaluation methods with different statistical metrics in each study

a. Methods validating/evaluating incidence										
Study	Evidence for data quality	Coverage of data source	Clear definition of incidence	Years of validation ≥ 10 yrs	Observed/reference data for validation	Statistical metric/indicator for validation	Methods sufficiently described to enable them to be replicated	Missing data assessment	Statistical software information	Total
Moller 2003	2	2	2	1	2	2	2	0	0	13
Pickle 2007	2	2	1	1	3	1	2	1	0	13
Mitton 2011	0	1	2	1	1	2	2	0	0	9
Rutherford 2012	2	1	0	2	2	2	2	0	0	11
Zhu 2012	2	2	0	1	3	1	2	1	0	12
Clèries 2012	2	2	0	2	2	2	0	0	0	10
Uhry 2013	0	2	2	1	2	1	0	0	0	8
Uhry 2013	0	2	1	1	2	2	0	0	0	8
Katanoda 2014	2	1	2	1	2	2	2	0	0	12
Antoni 2016	1	2	2	1	2	1	2	0	0	11
Poirier 2019	1	1	2	2	0	0	0	0	1	6
Earnest 2019	0	1	2	2	2	2	2	0	0	11
Demers 2020	0	1	1	1	2	2	2	0	0	9
Knoll 2020	2	1	2	2	2	2	2	0	1	13
Uhry 2020	0	2	2	1	2	2	2	0	1	11
Cheng 2021	2	1	0	2	1	2	2	0	0	10
Redondo-Sanchez 2021	2	1	2	2	2	2	2	0	0	13
Liu 2021	1	2	1	2	3	2	2	1	0	14
Luo 2021	0	1	0	2	2	2	2	0	0	9
Miller 2021	1	2	1	1	3	2	2	1	0	13
Li 2022	0	1	0	1	2	1	0	0	0	5
Luo 2022	0	1	2	2	2	1	2	0	0	10
Nguyen 2022	2	0	2	2	2	2	0	0	0	10
Bouzon Nagem Assad 2024	0	1	2	1	2	1	0	0	0	7

**b. Methods validating/evaluating prevalence**										
**First author and year**	**Evidence for data quality**	**Coverage of data source**	**Clear definition of prevalence**	**Years of validation ≥ 10 yrs**	**Observed/reference data for validation**	**Statistical metrics/indicator for validation**	**Methods sufficiently described to enable them to be replicated**	**Missing data assessment**	**Statistical software information**	**Total**
Mariotto 2006	0	2	1	1	2	0	2	0	1	9
Demuru 2023	0	2	1	1	2	2	0	0	1	9
Francisci 2023	0	2	1	1	2	1	2	0	1	10

**c. Methods validating/evaluating the number of treatment eligible patients**										
**First author and year**	**Evidence for data quality**	**Coverage of data source**	**Clear definition of treatment eligible patients**	**Years of validation ≥ 10 yrs**	**Observed/reference data for validation**	**Statistical metrics/indicator for validation**	**Methods sufficiently described to enable them to be replicated**	**Missing data assessment**	**Statistical software information**	**Total**
Campbell 2018	2	0	1	1	0	0	2	0	0	6
Nikolaou 2022	2	0	2	1	0	0	0	0	0	5

### Characteristics of studies reporting projection methods

The systematic search generated 176 records, with an additional 12 references obtained from supplemental sources. After excluding 21 duplicates, 167 references were screened for projection method, resulting in 26 articles that met the inclusion criteria for methods of NHL counts projection (Fig. 1b). Among these, 23 articles (88.5%) focused exclusively on incidence projection [14–16, 40, 43, 45, 66–82], one article (3.8%) projected prevalence alone [17], and two articles (7.7%) provided projections for both incidence, prevalence, and number of treatment eligible patients [6, 18].

Of the 26 eligible studies, 24 (92.3%) projected general NHL counts, while the remaining 2 (7.7%) projected the counts of DLBCL (Table 2b). No study projected the counts of MCL. Eighteen (68.2%) studies reported the statistical software used for NHL projection, with 7 studies using more than one statistical software in their projection methods. The most used software was R (30.8%), which included Nordpred, an R package developed for projection cancer cases in Nordic countries. Other commonly used software included Joinpoint (26.9%) and SEER (23.1%). Overall, 15 studies (57.7%) received a score of 8 or higher in a 10-point quality assessment, with three of those studies (11.5%) achieving a score of 10 by meeting all criteria for good quality (**Additional File 3c**). A detailed information of study characteristics is presented (**Additional File 4b**).

Among 25 studies estimating NHL incidence, nine distinct projection methods were identified (Table 5a). Eight studies used more than one method to project NHL incidence. The most common method projecting NHL incidence was age-period-cohort (APC) model (*n* = 11, 44%), a statistical method project disease incidence rates by considering the effects of age, period (time), and birth cohort. The second common projection method was joinpoint regression model (*n* = 8, 32%), a statistical method used to identify points where a significant change in the trend of a time series occurs. Additional projection methods including constant incidence rate assumption method (*n* = 5, 20%) and other generalized linear regression model, such as Poisson regression (*n* = 4, 16%), the state-space method (*n* = 2, 8%), and other four projection methods (*n* = 2, 8%).

**Table 5:** Summary of methods projecting NHL incidence, prevalence, and number of treatment eligible patients with NHL

Projection methods	References	Summary
**a) Methods projecting NHL incidence**		
Age-period-cohort (APC) model	Moller 2002 Mistry 2011 Nowatzki 2011 Zhu 2012 Uhry 2013 Rapiti 2014 Benhassine 2016 Donnelly 2020 Cameron 2021 Weir 2021 Asasira 2021	Incidence rates were modelled as a function of age, calendar period (year of age), and birth cohort (year of birth). The APC model was presented as: R_ap_ = (A_a_ + D · p + P_p_ + C_c_)^5^, where R_ap_ is the incidence rate in age group a in calendar period p, D is the common drift parameter accounting for the linear component of the trend in period and cohort, A_a_ is the age component for age group a, P_p_ is the non-linear period component of period p, and C_c_ is the non-linear cohort component of cohort c.
Joinpoint regression model	Pickle 2007 Zhu 2012 Shamseddine 2014 Donnelly 2020 Asasira 2022 Jung 2020 Jung 2021 Jung 2022	Joinpoint regression model is a non-linear regression model, which is comprised of a few continuous linear segments. The model is fitted by the least squares method for a given number of change points called joinpoints, and then the number of joinpoints is estimated. The tests of significance for change in trend use a Montre Cario Permutation method.
Constant incidence rate assumption method	Smith 2009 Rahib 2014 Rahib 2021 Kanas 2022 Sathishkumar 2022	A model assuming the average cancer incidence rate for the most recent years will remain constant into the future, or assuming that the observed annual percentage change in cancer rate will remain constant into the future.
Generalized linear model	Jung 2013 Jung 2014 Jung 2015 Dusek 2015 Kanas 2021	Poisson regression model or linear regression model.
Semiparametric Dirichlet process method (DIR)	Pickle 2007	A local linear model, in which the slope of the segment joining the number of deaths for any two consecutive time periods is assumed to be random with a nonparametric distribution, which has a Dirichlet process prior.
The previous ACS quadratic time series method	Pickle 2007	A model incorporates autocorrelation and a quadratic time trend, allowing for both linear and nonlinear changes over time.
The state-space method (SSM)	Pickle 2007 Zhu 2012	1. Assume a normal distribution for observed incidence counts at any point of time. 2. Model the average trajectory using a locally various quadratic trend. 3. Obtain the trend by representing the year-to-year variation of trend in the form of a so-called state-space model, whereby one relates the parameters (or state) of the model at a particular point to those of the previous point through a set of transition equations.
The Bayesian state-space (BSS) method	Zhu 2012	A model uses Bayesian inference to update beliefs about the states and parameters of the system given both the prior information and the observed data, allowing for more flexible and robust modeling of dynamic systems.
The vector autoregressive (VAR) model	Zhu 2012	A multivariate time series model capturing the relationships and dynamics among multiple variables over time. In a VAR model, each variable is regressed on its own lagged values as well as the lagged values of all other variables in the system.
**b) Methods projecting NHL prevalence**		
An epidemiological model incorporating NHL incidence and survival, and NHL recurrence	Dusek 2015	1. Estimate incidence using Poisson regression model. 2. Estimate survival using life-table methods.
An epidemiological model incorporating NHL incidence and survival	Kanas 2021	1. Estimate incidence based on constant incidence rate assumption. 2. Estimate the survival using the most recent observed annual survival rates.
An epidemiological model incorporating NHL incidence, survival, and likelihood and timing of cure	Chihara 2022	1. Estimate incidence based on historical incidence trend. 2. Estimate the survival of incident cohorts using survival curves corresponding to the calendar year of diagnosis (fitted by Kaplan Meier curves and Weibull parametric survival curve). 3. Characterization of cure: 1) earliest plausible year at which a proportion of patients were assumed to be cured (x1), proportion of surviving patients assumed to be cured at this time (p), and time point at which all surviving patients were assumed to be cured (x2).
**c) Methods projecting the number of treatment eligible patients with NHL**		
An epidemiological model incorporating NHL incidence, survival, NHL recurrence, and treatment pattern	Dusek 2015	1. Estimate incidence using Poisson regression model. 2. Estimate survival using life-table methods. 3. Estimate the recurrence based on information on patient’s health status and anti-tumor therapy applied after the one year following diagnosis 4. Estimate proportion of patients receiving anti-tumor therapy through cancer registries data
An epidemiological model incorporating NHL incidence and treatment pattern	Kanas 2021	1. Estimate incidence based on constant incidence rate assumption. 2. Estimate proportion of patients receiving systemic therapy through physician surveys

Among three studies estimating the prevalence of NHL, one focused on the prevalence of general NHL, while another two focused on the prevalence of DLBCL. All three studies calculated prevalence within limited time intervals by incorporating specific annual incidence rates and annual survival rates, with one study also considering the likelihood and timing of cure for DLBCL (Table 5b). Among two studies estimating the number of treatment-eligible patients with NHL, one estimated the counts of NHL by stage, and another one estimated the counts of DLBCL by LoT. No studies have reported estimated counts by treatment regimen. Both studies estimated the number of treatment-eligible patients by incorporating the incidence and treatment pattern, with one additional incorporating the survival and recurrence rates (Table 5c).

### Identifying robust methods for NHL cases estimation/projection

#### Evaluation of methods projecting the incidence of NHL

Four studies were excluded due to unavailable observed data for evaluation [70, 71, 74, 75]. Consequently, there were 19 studies with 18 projections of NHL incidence for male and female, and 17 studies with 11 projections of NHL incidence for both genders. The performance of these projections was evaluated and compared by country (Table 6). Six projections of NHL incidence were conducted in the US (methods 2a, 2b, 3, 6, 8, 14) [16, 40, 43, 66, 69]. In the projection by gender, the constant annual rate assumption method showed AARD and MARD < 10% for females in both studies (methods 8 and 14). However, this method demonstrated a higher AARD (12.7%) for males in one study only (method 8), while showing AARD < 10% in another study (method 14). In the total US population projections, a one-year projection using the joinpoint regression model (method 2a) demonstrated the lowest AARD value (1.6%), indicating that the joinpoint regression model was the most robust method projecting the incidence of NHL in the US. An additional projection of DLBCL incidence in the US was also validated, but the AARD for this projection was high (63.8%) (method 15).

**Table 6:** The summary AARD and MARD between observed and predicted NHL incidence counts

First author and publication year	No. methods	Country	NHL subtypes	Age period	Projection methods used	AARD (%)	MARD (%)
**Males**							
**Moller 2002**	1a	Denmark	NHL	Total	APC model	12.2%	20.2%
	1b	Finland				4.7%	15.6%
	1c	Iceland				8.4%	7.7%
	1d	Norway				5.6%	12.9%
	1e	Sweden				3.1%	7.9%
**Mistry 2011**	4	UK	NHL	10–24 yrs	APC model	53.5%	49.4%
				25–49 yrs		38.8%	38.6%
				50–64 yrs		2.4%	2.0%
				65–74 yrs		1.3%	1.3%
				75 + yrs		1.8%	2.0%
**Donnelly 2020**	12	UK	NHL	Total	APC model + Joinpoint regression model	6.8%	7.7%
**Nowatzki 2011**	5	Canada	NHL	Total	APC model	12.1%	13.8%
**Uhry 2013**	7a	France	NHL	Total	APC model + I/M ratio method	6.3%	6.4%
	7b				APC model + I/HD ratio method	6.3%	6.4%
	7c				APC model + I/LDD ratio method	6.5%	5.4%
**Rahib 2014**	8	USA	NHL	Total	Annual percentage change	12.7%	10.6%
**Rahib 2021**	14	USA	NHL	Total	Annual percentage change	6.2%	3.2%
**Rapiti 2014**	9	Switzerland	NHL	Total	APC model	15.2%	15.8%
**Shamseddine 2014**	10	Lebanon	NHL	Total	Joinpoint regression model	26.8%	20.6%
**Cameron 2021**	13	Australia	NHL	Total	Bayesian APC model	4.9%	–
**Jung 2013**	16	Korea	NHL	Total	Linear regression model	3.4%	2.0%
**Jung 2014**							
**Jung 2015**							
**Jung 2016**	17	Korea	NHL	Total	Joinpoint regression model	22.2%	26.9%
**Jung 2017**							
**Jung 2018**							
**Jung 2019**							
**Jung 2020**							
**Jung 2021**							
**Females**							
**Moller 2002**	1a	Denmark	NHL	Total	APC model	7.0%	15.0%
	1b	Finland			APC model	3.8%	19.5%
	1c	Iceland			APC model	20.9%	5.6%
	1d	Norway			APC model	6.4%	16.0%
	1e	Sweden			APC model	3.1%	12.7%
**Mistry 2011**	4	UK	NHL	25–49 yrs	APC model	37.1%	36.9%
				50–64 yrs		2.2%	1.6%
				65–74 yrs		2.2%	2.1%
				75 + yrs		1.7%	1.2%
**Donnelly 2020**	12	UK	NHL	Total	APC model + Joinpoint regression model	3.9%	1.9%
**Nowatzki 2011**	5	Canada	NHL	Total	APC model	10.6%	8.3%
**Uhry 2013**	7a	France	NHL	Total	APC model + I/M ratio method	5.8%	5.3%
	7b				APC model + I/HD ratio method	5.3%	4.0%
	7c				APC model + I/LDD ratio method	4.3%	1.3%
**Rahib 2014**	8	USA	NHL	Total	Annual percentage change	7.8%	5.2%
**Rahib 2021**	14	USA	NHL	Total	Annual percentage change	4.8%	2.3%
**Rapiti 2014**	9	Switzerland	NHL	Total	APC model	24.3%	25.2%
**Shamseddine 2014**	10	Lebanon	NHL	Total	Joinpoint regression model	32.9%	20.7%
**Cameron 2021**	13	Australia	NHL	Total	Bayesian APC model	4.9%	–
**Jung 2013**	16	Korea	NHL	Total	Linear regression model	4.2%	3.4%
**Jung 2014**							
**Jung 2015**							
**Jung 2016**	17	Korea	NHL	Total	Joinpoint regression model	36.3%	33.2%
**Jung 2017**							
**Jung 2018**							
**Jung 2019**							
**Jung 2020**							
**Jung 2021**							
**Both gender**							
**Pickle 2007**	2a	USA	NHL	Total	Joinpoint regression model	1.6%	–
	2b	USA	NHL	Total	Autoregressive quadratic time trend model	2.5%	–
**Smith 2009**	3	USA	NHL	Total	Constant incidence rate assumption	5.7%	3.6%
**Zhu 2012**	6	USA	NHL	Total	Joinpoint regression model	3.9%	–
**Rahib 2014**	8	USA	NHL	Total	Annual percentage change	10.9%	7.9%
**Rahib 2021**	14	USA	NHL	Total	Annual percentage change	5.0%	2.1%
**Kanas 2022**	15	USA	DLBCL	Total	Constant incidence rate assumption	63.8%	–
**Dusek 2015**	11	Czech Republic	NHL	Total	GLM	4.4%	–
**Cameron 2021**	13	Australia	NHL	Total	Bayesian APC model	5.2%	–
**Jung 2013**	16	Korea	NHL	Total	Linear regression model	1.9%	2.2%
**Jung 2014**							
**Jung 2015**							
**Jung 2016**	17	Korea	NHL	Total	Joinpoint regression model	7.8%	3.8%
**Jung 2017**							
**Jung 2018**							
**Jung 2019**							
**Jung 2020**							
**Jung 2021**							

NHL = non-Hodgkin lymphoma; AARD = average absolute relative deviation; APC = age-period-cohort; MARD = median absolute relative deviation; DLBCL = diffuse large B cell lymphoma; GLM = generalized linear model; I/M ratio = incidence/mortality; I/HD = incidence/hospital discharge; I/LDD = incidence/long-duration disease

Two projections were evaluated in the UK; one was the APC model across different age periods (method 4) [67] and another one was a combination of APC model and joinpoint regression model for the whole age period (method 12) [72]. The 10-year AARD of the APC model was less than 5% for males and females aged over 50 years, but increased up to 53.5% for the younger population. Meanwhile, the combination of APC model and joinpoint regression model displayed AARD and MARD < 10% for the whole age period, suggesting that this combination method was robust for projecting NHL incidence in the UK. In two projections for the Korean population [77–82], the AARD was 1.9% within 3 years using the linear regression model (method 16) and 7.8% within 6 years using the joinpoint regression model (method 17), suggesting that the latter was the most robust method projecting NHL incidence in Korea.

All remaining countries only employed one method in projecting NHL incidence. The APC model was evaluated in Nordic countries (methods 1a-1e), Canada (method 5), Switzerland (method 9), and Australia (method 13). The evaluation showed low AARD (< 10%) of APC model in Australia for both genders and in Iceland and Sweden males. However, the evaluation of joinpoint regression model in Lebanon (method 10) showed high AARD, with 26.8% in males and 24.3% in females.

#### Evaluation of methods projecting the prevalence of DLBCL

The performance of 29 projections from two studies estimating/projecting the DLBCL prevalence in the US was evaluated and compared (Table 7). Percent variations between the number of 21-yr prevalence cases at the year 2021 (2000–2021) derived by applying projection methods 1a-1ab and the number of 21-yr prevalence cases at the year 2021 estimated from SEER*Stat software were presented. In general, projection methods with cure and survival assumptions (methods 1a-1aa) produced lower values of 21-yr prevalence with respect to the estimation from SEER*Stat, ranging from 8.3% − 142.3%. When no cure was applied and all survivors were included for the duration of their lives, the projected 21-yr prevalence was moderately higher than the estimation from SEER*Stat (VAR% = −13.8%). Specifically, the VAR% of projection with assumptions of (1) the cured rate of 52.8%, (2) the cure begins at year 10 after diagnosis, and (3) all survival patients assumed to be cured at 20 years after diagnosis (method 1c) was closest to zero (VAR% = 8.3%), suggesting that this projection method might be the best one in the projection of DLBCL prevalence. In another study, compared to the observed 10-yr prevalence from SEER*Stat, the number of 10-yr DLBCL prevalence derived by applying the projection method 2 was 38.1% higher during 2020–2021.

**Table 7:** The summary percent variation between observed and predicted DLBCL prevalence counts

First author and publication year	No. methods	Country	NHL subtypes	Projection methods used	VAR%
Chihara 2022	1a	USA	DLBCL	52.8% cured rate + Cure begins at yr 1 + surviving patients assumed cured at yr 20	47.5%
	1b			52.8% cured rate + Cure begins at yr 5 + surviving patients assumed cured at yr 20	27.9%
	1c			52.8% cured rate + Cure begins at yr 10 + surviving patients assumed cured at yr 20	8.3%
	1d			52.8% cured rate + Cure begins at yr 1 + surviving patients assumed cured at yr 15	67.0%
	1e			52.8% cured rate + Cure begins at yr 5 + surviving patients assumed cured at yr 15	42.3%
	1f			52.8% cured rate + Cure begins at yr 10 + surviving patients assumed cured at yr 15	18.5%
	1 g			52.8% cured rate + Cure begins at yr 1 + surviving patients assumed cured at yr 10	105.2%
	1 h			52.8% cured rate + Cure begins at yr 5 + surviving patients assumed cured at yr 10	69.1%
	1i			52.8% cured rate + Cure begins at yr 10 + surviving patients assumed cured at yr 10	36.5%
	1j			67.2% cured rate + Cure begins at yr 1 + surviving patients assumed cured at yr 20	80.8%
	1k			67.2% cured rate + Cure begins at yr 5 + surviving patients assumed cured at yr 20	45.9%
	1 L			67.2% cured rate + Cure begins at yr 10 + surviving patients assumed cured at yr 20	15.6%
	1 m			67.2% cured rate + Cure begins at yr 1 + surviving patients assumed cured at yr 15	100.8%
	1n			67.2% cured rate + Cure begins at yr 5 + surviving patients assumed cured at yr 15	58.6%
	1o			67.2% cured rate + Cure begins at yr 10 + surviving patients assumed cured at yr 15	23.5%
	1p			67.2% cured rate + Cure begins at yr 1 + surviving patients assumed cured at yr 10	137.8%
	1q			67.2% cured rate + Cure begins at yr 5 + surviving patients assumed cured at yr 10	80.9%
	1r			67.2% cured rate + Cure begins at yr 10 + surviving patients assumed cured at yr 10	36.5%
	1s			68.9% cured rate + Cure begins at yr 1 + surviving patients assumed cured at yr 20	85.7%
	1t			68.9% cured rate + Cure begins at yr 5 + surviving patients assumed cured at yr 20	48.4%
	1u			68.9% cured rate + Cure begins at yr 10 + surviving patients assumed cured at yr 20	16.5%
	1v			68.9% cured rate + Cure begins at yr 1 + surviving patients assumed cured at yr 15	105.7%
	1w			68.9% cured rate + Cure begins at yr 5 + surviving patients assumed cured at yr 15	60.8%
	1x			68.9% cured rate + Cure begins at yr 10 + surviving patients assumed cured at yr 15	24.1%
	1y			68.9% cured rate + Cure begins at yr 1 + surviving patients assumed cured at yr 10	142.3%
	1z			68.9% cured rate + Cure begins at yr 5 + surviving patients assumed cured at yr 10	82.4%
	1aa			68.9% cured rate + Cure begins at yr 10 + surviving patients assumed cured at yr 10	36.5%
	1ab			No cure applied	−13.8%
Kanas 2021	2	USA	DLBCL	Incidence (year x) + Incidence (year x-1) × 1-year Survival + Incidence (year x-2) × 2-year Survival + …. + Incidence (year x-9) × 9-year Survival	−38.1%

NHL = non-Hodgkin lymphoma; DLBCL = diffuse large B cell lymphoma; VAR%=percent variation

#### Evaluation of methods projecting the number of treatment eligible patients with DLBCL or MCL by lot

Because of limited number and suboptimal quality of studies, no suitable method was found to evaluate methods estimating/projecting number of treatment eligible cancer patients. Therefore, the identification of the most appropriate method for forecasting the number of treatment eligible DLBCL or MCL patients by LoT was not employed in the present study.

## Discussion

This SLR characterized methods forecasting the incidence, prevalence, and number of treatment eligible patients with NHL by LoT. In addition, methods evaluating the performance of cancer forecasting methods were identified and utilized in selecting the most robust method applicable for projecting the incidence of NHL and prevalence of DLBCL in the US. In total, 29 studies reporting evaluation methods, and 26 studies reporting methods projecting the number of NHL cases were identified. AARD was identified as the most appropriate measurement for evaluating methods projecting NHL incidence. Furthermore, VAR% was chosen for the evaluation of methods projecting DLBCL prevalence. However, no appropriate method was found to evaluate projections of the number of treatment-eligible patients with DLBCL or MCL by LoT due to limited number and suboptimal quality of available methods. Our findings suggest that the joinpoint regression model might be the most robust method for projecting NHL incidence in the US. In addition, the projection based on following assumptions − 1) a 52.8% cure rate, 2) the onset of cure beginning 10 years after diagnosis, and 3) all survival patients assumed to be cured 20 years post-diagnosis - might be a robust method for projecting the prevalence of DLBCL. However, further research is needed to validate and project the number of treatment eligible patients by LoT for specific NHL subtypes, such as DLBCL and MCL.

### Methods to evaluate methods estimating/projecting cancer counts

There was no method for evaluating projection performance specifically in the context of NHL. To address this gap, we first conducted a SLR of evaluation methods in any cancer type, then performed a quality assessment to identify an appropriate method capable of evaluating the performance of NHL projection. The review showed that most evaluations were carried out for total or multiple cancer types, followed by evaluations for the specific cancers, such as lung and colorectal cancer [56, 62, 64]. In the evaluation of methods projecting cancer incidence, the selection of a method was heavily influenced by data availability. For example, three studies utilized SEER registries and the Cancer in North America (CiNA) Deluxe database to adjust the observed incidence counts for expected delays in case reporting [26, 43, 57]; thus, these three papers evaluated the projection by comparing the delay-adjusted observed data with projected values. In contrast, the majority of other studies evaluated projections through a direct comparison between observed data and projected values. In the assessment of evaluation methods, the AARD was identified as the most appropriate one to assess the accuracy of cancer incidence projection, with the advantage of avoiding a division of zero by adding an arbitrary small positive constant to the observed/delay-adjusted observed values in the denominator.

Only a limited number of studies have evaluated the performance of methods projecting cancer prevalence and the number of treatment eligible cancer patients. Among three studies that assessed the performance of methods projecting cancer prevalence, one study simply mentioned a comparison between projected prevalence and observed data without indicating any statistical metric [62], while the other two calculated the percent relative difference, or called percent variation [38, 63]. The quality of methods evaluating cancer prevalence projection was lower than methods evaluating cancer incidence projection (average quality score: 9.3 vs.10.3). All three studies reporting methods for prevalence evaluation did not report the quality of database and did not assess missing data. The VAR% used in Francisci et al. [38] was better than evaluation methods reported in other two studies because of the reporting of statistical indicator and a sufficiently description of the statistical method. In two articles evaluating methods projecting the number of treatment-eligible cancer patients, comparisons were made with earlier studies or report (e.g., 2012 worldwide cancer incidence and mortality estimated by WHO) and real-world sources (e.g., Kantar database) to evaluate the projection results [64, 65].

### Methods for NHL cases estimation/projection

To the best of our knowledge, this is the first study offering a systematic review and summary of methods projecting the incidence, prevalence, and number of treatment eligible patients by LoT with NHL, with a specific focus on DLBCL and MCL. Robust data exists regarding methods projecting incidence of NHL, with the APC model being the most frequently used method in European countries and Canada [14, 15, 45, 67, 68, 72–74]. This model allows for the investigation of how NHL incidence rates change over time and how these changes may be attributed to age, time period, or birth cohort. While the APC model is a valuable tool in cancer incidence projection, many studies have not taken advantage of APC model due to the concern about statistical identifiability and uncertainty of APC parameters interpretation [83]. In contrast to the APC model, the joinpoint regression model was more commonly used in projecting the incidence of NHL in the US [40, 43]. In a validation study, the joinpoint regression provided projections closest to the observed number of incident cases for NHL, indicating its effectiveness in capturing the trend changes for this cancer type [40]. In addition to the joinpoint regression model, the constant incidence rate assumption method was also widely used in NHL incidence projection in the US [6, 16, 66, 69]. This model is straightforward and assumes that the age-, sex-, and race-specific cancer incidence rates will remain constant over time. However, this method does not account for potential changes over time and is not able to reflect the change in risk factors and screening activities.

This review identified one study estimating the NHL prevalence in Czech Republic and two studies estimating DLBCL prevalence in the US [6, 17, 18]. All three studies constructed epidemiological models by integrating incidence and survival data from cancer registries or administrative claims databases to estimate prevalence cases. These models incorporated multiple assumptions of survival rates to represent potential population growth or medical advancement. For example, one study estimating the DLBCL prevalence in the US incorporated the concept of cure, defined as patients whose relapse-free survival extends beyond five years, and used various scenarios to account for different cure rates and timings [17]. In addition to estimate the prevalence of NHL, these model-based methods are consistently used in the literature to estimate or forecast the prevalence of other cancer types, such as colorectal carcinoma [62]. However, a primary limitation of this model is the lack of detailed disease status and treatment data over time in the database, necessitating assumptions about long-term cure versus progression to relapsed or refractory disease.

This review also identified one study estimating the number of NHL patients receiving anti-tumor therapy and another study estimating the number of treatment eligible patients with DLBCL by LoT [6, 18]. In projecting the number of patients with DLBCL, the patient count model additionally incorporated data from peer-reviewed literature and physician surveys to gather information on treatment pattern (e.g., proportion of new diagnosed patients inviting systemic therapy) [6]. Similarly, the patient count models used to estimate the number of patients with multiple myeloma and advanced non-small cell lung cancer by LoT also utilized treatment pattern data collected from literature reviews [64, 65]. However, collecting treatment information through physician surveys is restricted to the specific database, and may introduce bias due to the limited representativeness of the surveyed physicians. Further research is warranted to understand factors contributing to the survival curve, treatment pattern, and the proportion of untreated patients, especially in later LoTs for DLBCL.

### Identifying robust methods for NHL cases estimation/projection

The evaluation of the performance of NHL projection methods suggested huge variations in the projected number of NHL incidence cases. It is worth mentioning that the validation period for each incidence projection differs because of varying lengths of observation data available for evaluation in different countries or regions. For instance, the study forecasted the NHL incidence in Australia from 2017 to 2050, while the most recent observed NHL incidence data for Australia was in 2017 [36, 73]. Thus, the one-year AARD was calculated to evaluate the performance of Bayesian APC model projection method used in this study. In contrast, the 12-year AARD was calculated to evaluate the performance of the constant incidence rate assumption method used in the US. This is because the study forecasted the NHL incidence for 20 years from 2010 [69], and the latest available observation data in the US was in year 2021 [28]. These differences in validation periods may partially explain the huge variation in AARDs across studies. To address the impact of varying validation periods, we compared AARDs by country. In the US, among methods projecting NHL incidence, the joinpiont regression model showed the smallest AARD between observed and projected number of incident cases, suggesting that this model most accurately forecasted the incidence of NHL and might be the most robust projection method for NHL incidence. It is interesting that the most robust method projecting NHL incidence is different across countries. Although the joinpoint regression model performed well in incidence projection in the US, this method showed poorer estimation for the incidence projection in Lebanon and Korea. Instead, it was observed that the linear regression model projected incidence with smaller AARD and MARD than the joinpoint regression model in Korea, suggesting that the linear regression model might be a more robust method projecting NHL incidence in Korea. Further validation of diverse projection methods for the same period should be pursued to enhance their applicability and accuracy for projecting NHL incidence in the different countries.

Because of the limited number of studies projecting the prevalence of NHL identified in literature review, along with limited observational data sources, we focused our evaluation solely on the projected number of DLBCL prevalence in the US. The validation suggested that one projection method performed better than others. This method was based on assumptions including a 52.8% cure rate, the initiation of cure begins ten years after diagnosis, and the assumption that the remaining 47.2% of patients who are not initially defined as cured but survived beyond ten years after diagnosis would remain DLBCL patients until 20 years after diagnosis. Previous literature suggested that more than 60% of patients with DLBCL could be cured with first line therapy, and approximately 10–15% present with primary refractory disease and an additional 20–25% will relapse within the first 2 years of diagnosis [6]. In practice, it is unlikely for patients with uncured DLBCL, an aggressive subtype of NHL, to survive as long as 20 years. A real-world study highlighted that the median real-world OS was not reached for DLBCL patients with first line therapy during a median follow-up period of 28-month from diagnosis [84]. However, there was poor real-world OS among DLBCL patients who received subsequent LoTs, with survival of less than three years for the second LoT and less than one year for the third LoT [84]. The finding suggested that DLBCL patients with relapsed or refractory disease experienced suboptimal outcomes. Therefore, the assumption that the cure begins at 10 years after diagnosis and patients are considered cured at 20 years post-diagnosis may overestimate the actual number of DLBCL prevalence.

### Strengths and limitations

Our study has several strengths. It is the first comprehensive systematic review that synthesized evidence of methods evaluating the performance of cancer forecasting methods. We introduced a modified quality assessment tool to identify the most appropriate method for the evaluation of NHL projection methods. In addition, it is the first comprehensive systematic review that synthesized evidence on methods used to forecast NHL cases and identified the robust method to forecast the incidence of NHL and prevalence of DLBCL in the US. The evaluation of projection methods in our study provided an objective assessment of the agreement between newly released observed values and previously published projections. Furthermore, the accuracy of different projections in diverse demographics and cancer types would be evaluated as well by using our identified methods.

The study also has some limitations. First, a single reviewer screening strategy may raise concerns about the potential risk of missing relevant studies and inconsistencies in review process. However, a previous assessment has indicated that using a single reviewer in the SLR can maintain sensitivity and is acceptable [85]. To mitigate these risks, our single reviewer conducted two rounds of title/abstract screening: first using web app “Covidence” and then manually screening records. The high agreement rate for screening in aim 1, indicated by a Cohen’s κ value of 90%, further suggested the reliability of the screening process. Second, all studies identified in this review were unable to evaluate the impact of the COVID-19 pandemic on cancer estimation due to a 2–3 year lag in data collection and the lack of mature data on this matter. This situation may be compounded by publication bias, potentially influencing the true incidence, prevalence, and number of treatment eligible patients by LoT with NHL in the post-COVID-19 era. Third, while the criteria for assessing the quality of evaluation methods had not been previously validated and was firstly introduced in this study, it was designed based on the GATHER guidelines [25] and general principles of article quality, thereby ensuring the reliable assessment of evaluation methods. Fourth, as our study compared the performance of several projection methods, the evaluation period for each projection differed due to varying lengths of observation data available for evaluation, ranging from 1 to 15 years. To fully understand the reliability and accuracy of these projection methods, it is necessary to consider the impact of varying evaluation periods on their performance.

## Conclusion

In this SLR, we documented AARD and VAR% as the most appropriate measurement for evaluating methods projecting NHL incidence and prevalence, respectively. The evaluation of NHL projection methods has highlighted significant variability in the projected number of NHL cases, with different projection methods showing varying accuracy across countries. The joinpoint regression model was the most robust method for projecting NHL incidence in the US, while a specific projection method with assumptions of a 52.8% cure rate, a cure starting ten years post-diagnosis, and full cure at 20 years post-diagnosis was the most robust method for projecting DLBCL prevalence. There was no robust method forecasting the number of treatment eligible DLBCL or MCL patients by LoT. Future studies are needed to develop a valid method to estimate or forecast treatment eligible patients with NHL by LoT.

## Supplementary Information

Supplementary Material 1

## Data Availability

No datasets were generated or analysed during the current study.

## Abbreviations

AARD: Average absolute relative deviation
ADC: Antibody-drug conjugates
AIHW: Australian Institute of Health and Welfare
APC: Age-period-cohort
CAR-T: Chimeric antigen receptor T-cell therapy
CiNA: Cancer in North America
COVID-19: Coronavirus disease 2019
DLBCL: Diffuse large B cell lymphoma
FRANCIM: French Network of Cancer Registries
GATHER: Guidelines for Accurate and Transparent Health Estimates Reporting
ICD-O-3: International Classification of Disease for Oncology, 3rd Edition
LoT: Line of therapy
IRAC: International Agency for Research on Cancer
KNCI DB: Korea National Cancer Incidence Database
MARD: Median absolute relative deviation
MCL: Mantle cell lymphoma
mAbs: Monoclonal antibodies
NHL: Non-Hodgkin lymphoma
NPCR: National Program of Cancer Registries
PRISMA: Systematic Reviews and Meta-Analyses
SDMO: Study Design, Data, Methods, and Outcomes
SLR: Systematic literature reviews
US: United States
VAR%: Percent variation
