# Supplementary material for: Evaluation and forecasting methods to estimate number of patients with non-Hodgkin lymphoma: a systematic literature review
Source: Popul Health Metr. 2025 Oct 21;23:57. doi: 10.1186/s12963-025-00415-8 (PMC12539154; doi:10.1186/s12963-025-00415-8)
Supplement: Supplementary file 1 — Supplementary Material 1 [file 12963_2025_415_MOESM1_ESM.docx]

**Additional file 1.** SDMO criteria for studies reporting a) evaluation methods, and b) projection methods.

|  | **Inclusion Criteria** | **Exclusion Criteria** |
| --- | --- | --- |
| **a) Evaluation methods** | | |
| **S**tudy design | Noninterventional study Method/model assessment study Method/model evaluation study Method/model validation study Evidence synthesis study | Any not listed as inclusion |
| **D**ata | Data included incidence/prevalence/number of treatment eligible patients with any cancer in the national or regional level | Data did not include incidence/prevalence/number of treatment eligible patients with any cancer |
| **M**ethods | Method/model assessment Method/model evaluation Method/model validation Sensitivity analysis for method/model evaluation or validation | Any not listed as inclusion |
| **O**utcomes | Outcomes that assessed the goodness-of-fit/performance of projection methods, e.g., mean absolute percentage error (MAPE) | Outcomes that did not assess the goodness-of-fit/performance of projection methods |
| Other | Language: Literature published in English Population: Any cancer (excluding non-melanoma skin cancer) population Time: Literature published from Jan 1, 2002 to May 31, 2024 | Language: Literature did not publish in English Population: Non-cancer population Time: Literature published before Jan 1, 2002 or after May 31, 2024 |
| **b) Projection methods** | | |
| **S**tudy design | Noninterventional study Methodology study Evidence synthesis study | Any not listed as inclusion |
| **D**ata | Any population-based data includes incidence/prevalence/number of treatment eligible patients with NHL, specifically DLBCL and MCL by LoT | Data did not include incidence/prevalence/number of treatment eligible patients with NHL |
| **M**ethods | Projection method/model used to estimate or forecast the incidence/prevalence/number of treatment eligible patients with NHL, specifically DLBCL and MCL by LoT | Any not listed as inclusion |
| **O**utcomes | Cancer incidence Cancer prevalence Number of treatment eligible patients with NHL, specifically DLBCL and MCL by LoT | Any not listed as inclusion |
| Other | Language: Literature published in English Population: NHL cancer population Time: Literature published from Jan 1, 2002 to May 31, 2024 | Language: Literature did not publish in English Population: Non-NHL cancer population Time: Literature published before Jan 1, 2002 or after May 31, 2024 |

**Additional file 2.** Search strategy for studies reporting a) evaluation methods, and b) projection methods.

| **No.** | **Criteria** | **Strings** |
| --- | --- | --- |
| **a) Evaluation methods** | | |
| 1 | Forecasting filter | 'predictive model'/exp OR 'predictive model' OR 'forecasting model'/exp OR 'forecasting model' OR 'projection model' OR 'estimation model' OR 'population projection'/exp OR 'population projection' OR 'population growth estimation'/exp OR 'population growth estimation' OR 'forecasting'/exp OR 'forecasting' OR 'forecast' OR 'projection'/exp OR 'projection' OR 'temporal projection' OR 'projection methods' OR 'estimation'/exp OR 'estimation' OR 'estimating' OR 'projected trend' OR 'time series forecasting' |
| 2 | Evaluation filter | 'measurement accuracy'/exp OR 'measurement accuracy' OR 'predictive validity'/exp OR 'predictive validity' OR 'validation study'/exp OR 'validation study' OR 'standardization method' OR 'standard evaluation method' OR 'reliability'/exp OR 'reliability' OR 'accuracy'/exp OR 'accuracy' OR 'validity'/exp OR 'validity' OR 'validation'/de OR 'validation' OR 'sensitivity analysis'/de OR 'sensitivity analysis' OR 'assessment' OR 'evaluation' OR ‘robust method’ |
| 3 | Incidence/prevalence/number of treatment eligible patients filter | 'inciden*':ti OR 'prevalen*':ti OR 'treatment eligible patients':ab,ti OR 'line of therapy':ab,ti OR 'disease burden':ab,ti OR 'burden of disease':ab,ti OR 'epidemiology':ti OR 'cancer surveillance':ab,ti |
| 4 | Cancer filter | 'cancer'/exp OR 'cancer' OR 'carcinoma'/exp OR 'carcinoma' OR 'neoplasm'/exp OR 'neoplasm' OR 'malignancy'/exp OR 'malignancy' OR 'oncology'/exp OR 'oncology' OR 'non-hodgkin lymphoma'/exp OR 'non-hodgkin lymphoma' OR 'diffuse large b cell lymphoma'/exp OR 'diffuse large b cell lymphoma' OR 'mantle cell lymphoma'/exp OR 'mantle cell lymphoma' |
| 5 |  | #1 AND #2 AND #3 AND #4 |
| 6 | Limits | #5 AND [english]/lim AND [humans]/lim AND [2002-2024]/py |
| **b) Projection methods** | | |
| 1 | Forecasting filter | 'predictive model'/exp OR 'predictive model' OR 'forecasting model'/exp OR 'forecasting model' OR 'projection model' OR 'estimation model' OR 'population projection'/exp OR 'population projection' OR 'population growth estimation'/exp OR 'population growth estimation' OR 'forecasting'/exp OR 'forecasting' OR 'forecast' OR 'projection'/exp OR 'projection' OR 'temporal projection' OR 'projection methods' OR 'estimation'/exp OR 'estimation' OR 'estimating' OR 'projected trend' OR 'time series forecasting' |
| 2 | Incidence/prevalence/number of treatment eligible patients filter | 'inciden*':ti OR 'prevalen*':ti OR 'treatment eligible patients':ab,ti OR 'line of therapy':ab,ti OR 'disease burden':ab,ti OR 'burden of disease':ab,ti OR 'epidemiology':ti OR 'cancer surveillance':ab,ti |
| 3 | NHL filter | 'non-hodgkin lymphoma'/exp OR 'non-hodgkin lymphoma' OR 'diffuse large b cell lymphoma'/exp OR 'diffuse large b cell lymphoma' OR 'mantle cell lymphoma'/exp OR 'mantle cell lymphoma' |
| 4 |  | #1 AND #2 AND #3 |
| 5 | Limits | #4 AND [english]/lim AND [humans]/lim AND [2002-2024]/py |

**Additional file 3.** The quality appraisal checklist and results of quality assessment.

**a) Quality appraisal checklist**

| **Item** | **Yes** | **No** | **Unclear** |
| --- | --- | --- | --- |
| 1) Were the aims/objectives of the study clear? |  |  |  |
| 2) Was the study design appropriate for the stated aim? |  |  |  |
| 3) Was the sample size adequate? |  |  |  |
| 4) Were the study subjects and setting described in detail? |  |  |  |
| 5) Were objective, standard criteria used for the identification of the outcome? (e.g., outcomes were assessed based on existing diagnostic criteria, or self-reported scales?) |  |  |  |
| 6) Were outcome variables measured correctly using instruments/measurements that had been trialed, piloted, or published previously? |  |  |  |
| 7) Was there an appropriate statistical analysis? |  |  |  |
| 8) Is it clear what was used to determine statistical significance and/or precision estimates? (e.g., p values, CIs)? |  |  |  |
| 9) Were the methods (including statistical methods) sufficiently described to enable them to be repeated? |  |  |  |
| 10) Were the limitations of the study discussed? |  |  |  |

**b) Quality assessment of studies reporting evaluation methods**

| **First author and year** | **Clear aim and objectives** | **Appropriate study design** | **Adequate sample size** | **Description of study subjects and data source** | **Objective criteria to identify the outcome** | **Outcome variables were measured correctly using instruments/measurements that had been published previously** | **Appropriate statistical analysis** | **Determine precision estimates** | **Methods sufficiently described to enable them to be replicated** | **Limitations of the study discussed** | **Total** |
| --- | --- | --- | --- | --- | --- | --- | --- | --- | --- | --- | --- |
| Moller 2003 | ✓ | ✓ | ✓ | ✓ | ✓ | × | ✓ | × | ✓ | × | 7 |
| Pickle 2007 | ✓ | ✓ | ✓ | ✓ | ✓ | × | ✓ | × | ✓ | ✓ | 8 |
| Mitton 2011 | ✓ | ✓ | ✓ | ✓ | ✓ | ✓ | ✓ | × | ✓ | ✓ | 9 |
| Rutherford 2012 | ✓ | ✓ | ✓ | ✓ | × | ✓ | ✓ | ✓ | ✓ | × | 8 |
| Zhu 2012 | ✓ | ✓ | ✓ | ✓ | ✓ | × | ✓ | × | ✓ | ✓ | 8 |
| Clèries 2012 | ✓ | ✓ | ✓ | ✓ | × | ✓ | ✓ | ✓ | × | × | 7 |
| Uhry 2013 | ✓ | ✓ | ✓ | ✓ | × | ✓ | ✓ | ✓ | × | ✓ | 8 |
| Uhry 2013 | ✓ | ✓ | ✓ | ✓ | ✓ | ✓ | ✓ | ✓ | × | ✓ | 9 |
| Katanoda 2014 | ✓ | ✓ | ✓ | ✓ | ✓ | ✓ | ✓ | ✓ | ✓ | ✓ | 10 |
| Antoni 2016 | ✓ | ✓ | ✓ | ✓ | ✓ | ✓ | ✓ | × | ✓ | ✓ | 9 |
| Poirier 2019 | ✓ | ✓ | ✓ | ✓ | ✓ | × | ✓ | × | × | × | 6 |
| Earnest 2019 | ✓ | ✓ | ✓ | ✓ | ✓ | × | ✓ | ✓ | ✓ | ✓ | 9 |
| Demers 2020 | ✓ | ✓ | ✓ | ✓ | ✓ | ✓ | ✓ | × | ✓ | ✓ | 9 |
| Knoll 2020 | ✓ | ✓ | ✓ | ✓ | ✓ | × | ✓ | ✓ | ✓ | × | 8 |
| Uhry 2020 | ✓ | ✓ | ✓ | ✓ | × | ✓ | ✓ | ✓ | ✓ | × | 8 |
| Cheng 2021 | ✓ | ✓ | ✓ | ✓ | × | ✓ | ✓ | ✓ | ✓ | × | 8 |
| Redondo-Sanchez 2021 | ✓ | ✓ | ✓ | ✓ | ✓ | ✓ | ✓ | ✓ | ✓ | ✓ | 10 |
| Liu 2021 | ✓ | ✓ | ✓ | ✓ | ✓ | ✓ | ✓ | × | ✓ | ✓ | 9 |
| Luo 2021 | ✓ | ✓ | ✓ | ✓ | × | ✓ | ✓ | × | ✓ | ✓ | 8 |
| Miller 2021 | ✓ | ✓ | ✓ | ✓ | ✓ | ✓ | ✓ | × | ✓ | ✓ | 9 |
| Li 2022 | ✓ | ✓ | ✓ | ✓ | × | × | ✓ | × | × | ✓ | 6 |
| Luo 2022 | ✓ | ✓ | ✓ | ✓ | ✓ | ✓ | × | ✓ | ✓ | ✓ | 9 |
| Nguyen 2022 | ✓ | ✓ | ✓ | × | ✓ | ✓ | ✓ | × | × | ✓ | 7 |
| Bouzon Nagem Assad 2024 | ✓ | ✓ | ✓ | ✓ | ✓ | × | ✓ | × | × | × | 6 |
| Mariotto 2006 | ✓ | ✓ | ✓ | ✓ | ✓ | ✓ | ✓ | × | ✓ | × | 8 |
| Demuru 2023 | ✓ | ✓ | ✓ | ✓ | ✓ | ✓ | ✓ | × | ✓ | ✓ | 9 |
| Francisci 2023 | ✓ | ✓ | ✓ | ✓ | ✓ | ✓ | ✓ | ✓ | ✓ | ✓ | 10 |
| Campbel 2018 | ✓ | ✓ | ✓ | ✓ | × | × | ✓ | ✓ | ✓ | ✓ | 8 |
| Nikolaou 2022 | ✓ | ✓ | ✓ | × | × | × | ✓ | ✓ | × | ✓ | 6 |

**c) Quality assessment of studies reporting projection methods**

| **First author and year** | **Clear aim and objectives** | **Appropriate study design** | **Adequate sample size** | **Description of study subjects and data source** | **Objective criteria to identify the outcome** | **Outcome variables were measured correctly using instruments/measurements that had been published previously** | **Appropriate statistical analysis** | **Determine precision estimates** | **Methods sufficiently described to enable them to be replicated** | **Limitations of the study discussed** | **Total** |
| --- | --- | --- | --- | --- | --- | --- | --- | --- | --- | --- | --- |
| Moller 2002 | ✓ | ✓ | ✓ | ✓ | ✓ | ✓ | ✓ | ✓ | ✓ | × | 9 |
| Pickle 2007 | ✓ | ✓ | ✓ | ✓ | ✓ | ✓ | ✓ | × | ✓ | ✓ | 9 |
| Smith 2009 | ✓ | ✓ | ✓ | ✓ | ✓ | ✓ | ✓ | × | ✓ | × | 8 |
| Mistry 2011 | ✓ | ✓ | ✓ | ✓ | ✓ | ✓ | ✓ | × | ✓ | × | 8 |
| Nowatzki 2011 | ✓ | ✓ | ✓ | × | ✓ | ✓ | ✓ | × | ✓ | ✓ | 8 |
| Zhu 2012 | ✓ | ✓ | ✓ | ✓ | ✓ | ✓ | ✓ | ✓ | ✓ | ✓ | 10 |
| Uhry 2013 | ✓ | ✓ | ✓ | ✓ | ✓ | ✓ | ✓ | ✓ | × | ✓ | 9 |
| Rahib 2014 | ✓ | ✓ | × | × | × | × | ✓ | × | × | × | 3 |
| Rapiti 2014 | ✓ | ✓ | ✓ | ✓ | ✓ | ✓ | ✓ | × | ✓ | ✓ | 9 |
| Shamseddine 2014 | ✓ | ✓ | × | × | ✓ | × | ✓ | × | × | ✓ | 5 |
| Dusek 2015 | ✓ | ✓ | ✓ | ✓ | ✓ | ✓ | ✓ | ✓ | × | × | 8 |
| Benhassine 2016 | ✓ | ✓ | ✓ | ✓ | ✓ | × | ✓ | ✓ | × | × | 7 |
| Donnelly 2020 | ✓ | ✓ | ✓ | ✓ | ✓ | ✓ | ✓ | ✓ | ✓ | ✓ | 10 |
| Cameron 2021 | ✓ | ✓ | × | × | ✓ | ✓ | ✓ | ✓ | ✓ | ✓ | 8 |
| Rahib 2021 | ✓ | ✓ | ✓ | ✓ | ✓ | ✓ | ✓ | ✓ | ✓ | ✓ | 10 |
| Weir 2021 | ✓ | ✓ | ✓ | ✓ | ✓ | ✓ | ✓ | × | ✓ | ✓ | 9 |
| Asasira 2022 | ✓ | ✓ | ✓ | ✓ | ✓ | × | ✓ | × | ✓ | ✓ | 8 |
| Chihara 2022 | ✓ | ✓ | ✓ | ✓ | ✓ | ✓ | ✓ | × | ✓ | ✓ | 9 |
| Kanas 2022 | ✓ | ✓ | ✓ | ✓ | ✓ | × | ✓ | × | × | ✓ | 7 |
| Sathishkumar 2022 | ✓ | ✓ | ✓ | ✓ | ✓ | × | × | × | × | × | 5 |
| Jung 2013 | ✓ | ✓ | ✓ | ✓ | ✓ | ✓ | ✓ | × | × | × | 7 |
| Jung 2014 | ✓ | ✓ | ✓ | ✓ | ✓ | ✓ | ✓ | × | × | × | 7 |
| Jung 2015 | ✓ | ✓ | ✓ | ✓ | ✓ | ✓ | ✓ | × | × | × | 7 |
| Jung 2020 | ✓ | ✓ | ✓ | ✓ | ✓ | ✓ | ✓ | × | × | × | 7 |
| Jung 2021 | ✓ | ✓ | ✓ | ✓ | ✓ | ✓ | ✓ | × | × | × | 7 |
| Jung 2022 | ✓ | ✓ | ✓ | ✓ | ✓ | ✓ | ✓ | × | × | × | 7 |

**Additional file 4.** Detailed characteristics for studies reporting a) Evaluation methods, and b) Projection methods

| **a) Evaluation methods** | | | | | | | | |
| --- | --- | --- | --- | --- | --- | --- | --- | --- |
| **First Author** | **Year** | **Data source** | **Data coverage** | **Country** | **No. of years projected** | **No. of years validated/evaluated** | **Cancer site** | **Outcomes to be validated/evaluated** |
| **Bjorn Moller** | 2003 | Nordic cancer registries (1958-1997) | Multi-country | Denmark Finland Iceland Norway Sweden | 5 yrs | 5 yrs | 20 cancer sites (includes **NHL**) | Incidence |
| **Linda Pickle** | 2007 | 1. SEER 17 2. NAACCR (1995-2003) | Subnational | US | 1 yr | 1 yr | breast, prostate, lung and bronchus, colon and rectum), **NHL**, melanoma, and esophagus for 2001, all sites for 2007 | Incidence |
| **Nicolas Mitton** | 2011 | The hospital database of the Programme de Medicalisation des Systemes d’Information Medicale (PMSI) | National | France | 1 yr | 1 yr | colon-rectum, breast, ovary, and kidney cancers | Incidence |
| **Mark J. Rutherford** | 2012 | Finnish cancer registry data | Subnational | Finland | 20 yrs | 20 yrs | breast, lung, colon and pancreas cancers | Incidence |
| **Li Zhu** | 2012 | 1. Cancer in North America (CINA) Deluxe incidence data from NAACCR (1995-2007) 2. SEER 9 registries plus 2 additional area | Subnational | US | 2 yrs | 1 yr | Total cancer (includes **NHL**), by site, by gender | Incidence |
| **Ramon Clèries** | 2012 | Tarragona Cancer Registry (TCR) and Girona Cancer Registry (GCR) | Subnational | Spain | short term: 3-4 yrs Long-term: 10 yrs | short term: 3-4 yrs Long-term: 10 yrs | Breast cancer Colorectal cancer Lung cancer Prostate cancer | Incidence |
| **Zoe Uhry** | 2013 | French cancer registries | Subnational | France | 3 yrs | 3 yrs | 22 cancer sites (includes **NHL**), by site, by gender | Incidence |
| **Zoe Uhry** | 2013 | 1. French cancer registries 2. ALD data, the chronic disease scheme of the French national health insurance system | Subnational | France | 6 yrs | 6 yrs | 24 cancer sites (includes **NHL**), by site, by gender | Incidence |
| **Kota Katanoda** | 2014 | Population-based cancer registries in four prefectures (Miyagi, Yamagata, Fukui and Nagasaki), | Subnational | Japan | 5 yrs | 5 yrs | 7 cancer sites (stomach, liver, colon/rectum, lung, female breast, cervix uteri, and prostate) | Incidence |
| **Sebastien Antoni** | 2016 | NORDCAN (Nordic cancer) database | National | Norway | 1 yr | 1 yr | Total cancer (includes **NHL**), by site Men, by site Women, by site | Incidence |
| **Abbey E. Poirier** | 2019 | National Cancer Incidence Reporting System (NCIRS) for 1983-1991, Canadian Cancer Registry (CCR) for 1992-2012 | National | Canada | 30 yrs | 30 yrs | Colorectum Lung Bladder Breast Prostate | Incidence |
| **Arul Earnest** | 2019 | The 2013 version of the Australian Cancer Database (ACD) | National | Australia | 10 yrs | 10 yrs | Prostate cancer | Incidence |
| **Alain Demers** | 2020 | National Cancer Incidence Reporting System and Canadian Cancer Registry | Subnational (the province of Quebec was not included) | Canada | 4 yrs | 4 yrs | 24 cancer sites (includes **NHL**), by site, by gender | Incidence |
| **Maximilian Knoll** | 2020 | SEER-9,  NORDCAN,  and Saarland | Multi-country | US Denmark Finland Iceland Norway Sweden Faroe Islands and Greenland German | 10 yrs | 10 yrs | Three low incident tumor sites: brain tumors, kidney cancer, and melanoma; Four high incident entities: lung, breast, colorectal, prostate | Incidence |
| **Zoe Uhry** | 2020 | French cancer registry | Subnational | France | 19 yrs | 5 yrs | 22 cancer sites (includes **NHL**), by site, by gender female lung cancer and prostate cancer as examples | Incidence |
| **Fang Cheng** | 2021 | Global Burden of Disease (GBD) Study | National | China | 30 yrs | 25 yrs | Thyroid cancer | Incidence |
| **Daniel Redondo-Sanchez** | 2021 | Granada Cancer Registry | Subnational | Spain | 10 yrs | 10 yrs | **Total Cancer** (except for non-melanoma skin cancer)  **Men**: stomach, colon, rectal, lung, prostate, bladder, and other **Women**: colon, rectal, lung, breast, corpus uteri, ovarian, and other | Incidence |
| **Benmei Liu** | 2021 | Cancer in North America (CiNA) Deluxe incidence data from NAACCR (1996-2014) | Subnational | US | 19 yrs | 19 yrs | 47 (includes **NHL**) cancer sites, by site | incidence |
| **Qingwei Luo** | 2021 | -  (Lit review for projection methods) | Multi-country | Multi-country | ≥10 yrs | ≥10 yrs | Lung cancer | Incidence |
| **Kimberly Miller** | 2021 | CiNA Deluxe incidence data from NAACCR (1996-2014) | Subnational | US | 1 yr | 1 yr | 47 (includes **NHL**) cancer sites in total | incidence |
| **Jinhui Li** | 2022 | CI5plus, from the International Agency for Research on Cancer under the World Health Organization | Multi-country | 118 regions/countries (Table 1) | 3, 5, and 10 yrs, respectively | 3 yrs and 5 yrs respectively | Seven aging-related cancers (bladder, colorectal, esophagus, lung, pancreas, prostate, and stomach) | Incidence |
| **Qingwei Luo** | 2022 | Australian Institute of Health and Welfare (AIHW) | National | Australia | 25 yrs | 10 years | 21 cancer sites (incudes **NHL**) | Incidence |
| **Phuong The Nguyen** | 2022 | Cancer Statistics in Japan (CSJ) | Not reported | Japan | 35 yrs | 15 yrs | 22 cancer sites (includes **NHL**) | Incidence |
| **Daniel Bouzon Nagem Assad** | 2024 | Instituto Nacional do Câncer (INCA) | Subnational | Brazil | 39 yrs | 3 yrs | 7 cancer sites (breast, colorectal, prostate, lung, cervical, head and neck, and childhood) | Incidence |
| **Angela B. Mariotto** | 2006 | SEER-9 (1973-1999) | Subnational | US | 21 yrs | 3 yrs | Colorectal cancer | Prevalence |
| **Elena Demuru** | 2023 | 62 general cancer registries from 27 European countries (21 with national population coverage) | Multi-country | 27 European countries | - | 1 yr | Total cancer (include **NHL**), by site, by gender | Prevalence |
| **Silvia Francisci** | 2023 | Veneto Cancer Registry (VCR) | Subnational | Italy | 1 yr | 1 yr | colon and rectum, lung, and breast cancers | Prevalence |
| **David Campbel** | 2018 | United Nations (UN) world population data | Multi-country | France Germany Italy Spain | 5 yrs | 5 yrs | Non-small cell lung cancer (NSCLC) | Number of treatment eligible NSCLC patients by LoT (L2 and L3) |
| **Andreas Nikolaou** | 2022 | SEER and lit review | Subnational | US | 1 yr | 1 yr | Multiple myeloma | Number of treatment eligible MM patients by LoT (L1, L2, L3, L4, L5+) |
| **b) Projection methods** | | | | | | | | |
| **First Author** | **Year** | **Data source** | **Data coverage** | **Country** | **No. of years projected** | **With validation?** | **NHL subtypes** | **Outcomes** |
| **Bjorn Moller** | 2002 | The Nordic cancer registries | National | Nordic countries (Denmark, Finland, Iceland, Norway, and Sweden) | 25 yrs | No* | NHL | Incidence |
| **Linda Pickle** | 2007 | 1. SEER 17 (1988-1997) 2. NAACCR (1995-2003) | Subnational | US | 1 yr | Yes | NHL | Incidence |
| **Benjamin D. Smith** | 2009 | SEER-17 database | Subnational | US | 23 yrs | No | NHL | Incidence |
| **M. Mistry** | 2011 | UK Association of Cancer Registries (England, Scotland and Wales; 1975-2007) | Subnational | UK | 23 yrs | No | NHL | Incidence |
| **Janet Nowatzki** | 2011 | Manitoba Cancer Registry (1976-2005) | Subnational | Canada | 21 yrs | No | NHL | Incidence |
| **Li Zhu** | 2012 | 1. CINA Deluxe incidence data from NAACCR (1995-2007) 2. SEER 9 registries plus 2 additional area | Subnational | US | 1 yr | Yes | NHL | Incidence |
| **Zoe Uhry** | 2013 | French cancer registries | Subnational | France | 3 yrs | Yes | NHL | Incidence |
| **Lola Rahib** | 2014 | Not reported | Not reported | US | 20 yrs | No | NHL | Incidence |
| **Elisabetta Rapiti** | 2014 | National Institute for Cancer Epidemiology and Registration (NICER) | Subnational | Switzerland | 5 yrs | No* | NHL | Incidence |
| **Ali Shamseddine** | 2014 | National Cancer Registry database of the Ministry of Public Health in Lebanon | Not reported | Lebanon | 10 yrs | No | NHL | Incidence |
| **Ladislav Dusek** | 2015 | Czech National Cancer Registry (CNCR) | National | Czech Republic | 2 yrs | No | NHL | Incidence Prevalence Number of patients treated with anti-tumor therapy |
| **Adel Benhassine** | 2016 | North-Tunisia Cancer Registry (NTCR) | Subnational | Tunisia | 1 yr | No | NHL | Incidence |
| **David W. Donnelly** | 2020 | Northern Ireland Cancer Registry (NICR) | Subnational | UK | 23 yrs | No | NHL | Incidence |
| **Jessica Katherine Cameron** | 2021 | Australian Institute of Health and Welfare (AIHW) Australian Cancer Database | Not reported | Australia | 15 yrs | Yes | NHL | Incidence |
| **Lola Rahib** | 2021 | SEER 21 2000-2016 US Census Bureau | Subnational | US | 25 yrs | Yes | NHL | Incidence |
| **Hannah Weir** | 2021 | SEER 1975-2017 | Subnational | US | 36 yrs | No | NHL | Incidence |
| **Judith Asasira** | 2022 | Kampala Cancer Registry 2001-2015 | Subnational | Uganda | 15 yrs | No | NHL | Incidence |
| **Gena Kanas** | 2022 | 1. Country-specific cancer registries for the US, France, Germany, Italy, Spain, and the UK  2. Peer reviewed literature 3. Results of a physician survey conducted by Kantar Health | National | US France Germany Italy Spain UK | 6 yrs | No | DLBCL | Incidence Prevalence Number of treatment eligible DLBCL patients by LOT (L1, L2, L3+) |
| **Krishnan Sathishkumar** | 2022 | National Cancer Registry Program (NCRP) in India | Subnational | India | 1 yr | No | NHL | Incidence |
| **Kyu-Won Jung** | 2013 | Cancer Incidence Data: Korea National Cancer Incidence Database | National | Korea | 1 yr | No | NHL | Incidence |
| **Kyu-Won Jung** | 2014 |  |  |  | 1 yr |  | NHL | Incidence |
| **Kyu-Won Jung** | 2015 |  |  |  | 1 yr |  | NHL | Incidence |
| **Kyu-Won Jung** | 2020 |  |  |  | 1 yr |  | NHL | Incidence |
| **Kyu-Won Jung** | 2021 |  |  |  | 1 yr |  | NHL | Incidence |
| **Kyu-Won Jung** | 2022 |  |  |  | 1 yr |  | NHL | Incidence |
| **Dai Chihara** | 2022 | SEER 18 2000-2018 | Subnational | US | 1 yr | No | DLBCL | Prevalence |
